# On the Remedial Influence of Oxygen or Vital Air, Nitrous Oxide, and Other Gases, Electricity, and Galvanism, in Restoring the Healthy Functions of the Principal Organs of the Body and the Nerves Supplying the Respiratory, Digestive, and Muscular Systems

**Published:** 1845-01

**Authors:** 


### ART. XIX.

*On the Remedial Influence of Oxygen or Vital Air, Nitrous Oxyde, and other Gases, Electricity, and Galvanism, in restoring the healthy functions of the principal Organs of the Body and the Nerves supplying the Respiratory, Digestive, and Muscular Systems.* By J. EVANS RIADORE, M.D. F.L.S. &c.—*London*, 1845. 8vo, pp. 178.

THIS is another specimen, by the same author, of a class of books which we have always exposed and condemned—books written for the public under the professional cover, and written, we shall not say designedly and purposely to attract ignorant patients to the doors of their authors, but, most assuredly, written as cleverly as if this had been the express object of their composition. Liebig's theories are now the rage, and attracting the attention of the whole reading public, learned and unlearned. It was therefore very natural for one of the class of writers we have at present in view, to endeavour

"To share the triumph and partake the gale,"

by hooking on his miserable dirty little shallop to the stern of the magnificent bark now so proudly careering "the waves of glory."

"Professor Liebig's works on organic chemistry," writes Dr. Riadore, in his own lucid style, "have introduced a new *therapeutical branch* to the profession; and, consequently, increased a desire for a more intimate acquaintance with animal chemistry and its practical application, to correspond with the more philosophical method now adopted in investigating diseased conditions of the living system. I have endeavoured to apply, concisely, Liebig's theories," &c. (Pref. p. i.)

Nor was this a matter of mere choice; our modest author was really *forced* to make his patients inhale "oxygen *or vital air.*" How could a man of science and feeling do otherwise?

"Most of the patients," says Dr. Riadore, "*who were induced to consult me* THROUGH MY LATE WORK, had their maladies in the chronic form, having consulted, in many instances, some of the most eminent practitioners of the day, [a very unhandsome proceeding, by the way, on the part of the patients, to make our learned author the *pis-aller* of London,] and, consequently, I found it would be more than useless to pursue the ordinary mode of treatment. I, therefore, had recourse to untried means," &c. (Pref. p. vii.)

And it is to the exposition of these *untried* means, so kindly announced in its very explicit title-page, that this volume is devoted. This sort of title-page, by the way, will save us and other reviewers much trouble, as it contains nearly everything that the book contains or was intended to contain, and in more intelligible language than that of the volume itself. Intelligibility is not our author's *forte* certainly, though we are far from thinking that unintelligibility is his *foible ;* because we have a shrewd suspicion that the *ignotum pro magnifico* is the very kind of fare best suited for the palate of intended readers.

Of course, all obstinate diseases vanish under the battery of the new armamentarium of Harley-street—and, among these, of course, all such little ailments as consumption, and asthma, and sterility, and deafness, and blindness, &c. Sterility and its congeners are very favorite subjects with the author. Besides numerous incidental notices, we have one whole chapter on " disorders of the generative functions,"—a filthy subject filthily treated ; and we are soon to have a whole work on this precious theme from our philanthropic author. " I shall shortly publish," he says, " my experience, in a distinct treatise, on spermatorrhea, or involuntary emission, and other *defective functions* of the generative organs." What boundless blessings to the unhappy may be expected from a whole " distinct treatise," devoted to their service, when we have many such beatific revelations as the following, even in a single chapter:

" Gentlemen who live generously, and leading a sedentary life, are continually met with, complaining of heated skin, clammy white tongue, headach, acrid eructations, oppressed breathing, fickleness, irritability, or considerable despondency, amounting to a desire to seek death, with disinclination to all mental or physical occupation, and *with a very low degree, and even sometimes absence of animal passion.* Females are similarly affected, and this state is attended with considerable derangement of the uterine functions, or a *total loss of the generative function, or power of conception;* some fancy that they hear in their sleep, or even while awake, heavenly music, or a diabolical conversation, or recommendations, in a whisper, to destroy themselves. Patients of the above description are generally only functionally and not organically affected, and are capable of *being quickly either cured or greatly benefited,* by inhalation of oxygen, and the use of galvanism, and other simple remedies, and a judicious diet." (p. 74.)

" I have administered the oxygen in cases of sterility, with the utmost advantage. In cases of leucorrhea, which had resisted the usual remedial means, I have also found inhalation of the oxygen of much advantage, and believe that few of these cases may not be cured in the absence of organic causes, by oxygen, galvanism, and tonic medicines containing iron and ammonia. Two ladies, after having been married five and seven years, without having, I believe, ever conceived, although, as usual, they said they had several mishaps, became patients of mine, in consequence of intolerable sufferings from dysmenorrhea, and were not only cured by oxygen and electricity, but actually became pregnant, and gave birth to fine children, and one of the patients is again pregnant." (pp. 81-2.)

Consumption, as we have said, is also one of the favorite objects and one of the glorious monuments of the new treatment.

" From very extensive trials, for some years past, I am fully satisfied that some cases of consumption are remediable, and often curable, by inhalation of some of the gases, that would inevitably prove fatal by depending upon administering medicines by the stomach, according to the usual system." (p. 99.)

In the first of the cases given to show " the benign effect of the inhalation of oxygen," we are indebted to our learned author for some important novelties in physiology, pathology, and practice. " The animal heat," we are told was at first 100; "in four days was reduced to 98," and subsequently it was reduced "to its *natural standard*, 97°" !!

Asthma naturally follows consumption: "Asthma, depending upon functional derangements, will generally be removed or alleviated by inhalation of oxygen a few times." (p. 103.) Even stricture of the urethra cannot withstand the doctor's aerial battery: "In a case of spasmodic stricture of the urethra, I have used the nitrous oxyde gas with success." (p. 118.)

*Deafness.* "A gentleman consulted me respecting deafness, more particularly of the left ear, which followed a severe cold succeeding the measles. He had tried a great many means, usually adopted by aurists, without any benefit. I was induced to try the nitrous oxyde gas; and, under its first influence, he considered it improved his hearing. *I recommended him to come to me every day*, and I galvanised his ears in the usual way one day and administered the nitrous oxyde gas the following day. Thus, with a little alterative medicine, he was cured in six weeks, and continues well in every respect. This gentleman having suffered so long, and having a large circle of acquaintances, he recommended several deaf patients to consult me, and I *have pursued the same plan in all the cases that I regarded as dependent upon deficient nervous energy,* WITH THE UTMOST SATISFACTION." (pp. 119-20.)

We entertain no doubt whatever of "the satisfaction" derived from this *plan*. Is it not extremely satisfactory to see one's friends come to one "every day"—more especially, as our learned author would say, "dona ferentes?"

*Blindness.* "I can assure the members of *my profession,* [of course, no one else!] that the function of the optic nerves may be restored by a simple alterative course of medicine, and by inhalation of nitrous oxyde gas, and by electro-stimulation, after all the usual remedial means have been tried unsuccessfully." (p. 121.)

In a case of blindness from iritis, the result was, of course, happy:

"When I first saw this patient he could only see the waving of a hand or the reflection of a bright object. The vitreous humour was of a greenish colour, and the eye apparently fixed as it were upon vacuity. Both eyes were irritable, his general health was bad, and he felt very desponding. All these symptoms are now, however, *a matter of history,* [!!] and he is able to perform his duties as a clergyman." (pp. 122-3.)

But, as the author says "it would be useless to relate various cases of deafness and of blindness, arising from defective nervous energy of the respective nerves of the ear and eye, for in truth, the success attending the means have been most generally singularly gratifying," (p. 123;) so we must, however reluctantly, here terminate our very imperfect account of the mighty marvels contained in this book, which will justly entitle its author to an elevated rank among the curers of the incurable.

But before laying down our pen, we must add a word or two in a graver strain. In dissecting, for the instruction of our readers, such disgusting *subjects* as that whereof we have just interred the remains, we have, it is true, the satisfaction to know and feel that we have discharged a public duty. But, alas, we cannot flatter ourselves with the hope that we have thereby destroyed the infection wherewith they were imbued, so long as there remains a venal non-medical press to scatter it among the lieges. We do not doubt, that before the page we are now writing shall see the light, the very book we have here denounced will be lauded to the skies in some of our daily and weekly newspapers. In our Number for July last, we gave a notice of some books by an author of this class —a notice which we defy any one to prove unjust—remarking of them that "they prove the total incapacity of the author to write his mother tongue, and justify the inference that his scientific knowledge bears some proportion to his literary proficiency." And, then, we see, in a most influential daily paper, a review of one of the very same books, in the following strain:

"The design of the scientific author of this Treatise is obvious, and merits commendation. By describing in popular language subjects which have been too generally veiled, by technical and scientific phraseology, from those most interested in a knowledge of their character and consequences, Dr. —— has conferred a great benefit on the more delicate and retiring sex. For obvious reasons we cannot here enter into details. Our meaning must be rather implied than explained. But, without trespassing beyond our due limits, we may advise that this practical compendium—the result of Dr.——'s extensive observation and experience during a long course of practice as a physician—be possessed by every female and especially by every mother. Its clear and carefully-explained symptoms—a mistake respecting which is often attended by dangerous and even fatal results—and an attention to the salutary cautions and directions it furnishes, will prevent much anxiety, and lead to the recovery of health, even when the sufferer and her friends were anticipating protracted sickness, if not a fatal termination."

Can any of our readers inform us by whom such articles are written, and by what means their insertion in respectable newspapers is procured?

---

